# Handedness, language areas and neuropsychiatric diseases: insights from brain imaging and genetics

**DOI:** 10.1093/brain/awz257

**Published:** 2019-09-05

**Authors:** Akira Wiberg, Michael Ng, Yasser Al Omran, Fidel Alfaro-Almagro, Paul McCarthy, Jonathan Marchini, David L Bennett, Stephen Smith, Gwenaëlle Douaud, Dominic Furniss

**Affiliations:** 1 Nuffield Department of Orthopaedics, Rheumatology, and Musculoskeletal Science, University of Oxford, Oxford, UK; 2 Nuffield Department of Clinical Neurosciences, University of Oxford, John Radcliffe Hospital, Oxford, UK; 3 Centre for Functional MRI of the Brain (FMRIB), Wellcome Centre for Integrative Neuroimaging, University of Oxford, Oxford, UK; 4 Department of Statistics, University of Oxford, Oxford, UK

**Keywords:** handedness, microtubules, arcuate fasciculus, GWAS, Parkinson’s disease

## Abstract

Ninety per cent of the human population has been right-handed since the Paleolithic, yet the brain signature and genetic basis of handedness remain poorly characterized. Here, we correlated brain imaging phenotypes from ∼9000 UK Biobank participants with handedness, and with loci found significantly associated with handedness after we performed genome-wide association studies (GWAS) in ∼400 000 of these participants. Our imaging–handedness analysis revealed an increase in functional connectivity between left and right language networks in left-handers. GWAS of handedness uncovered four significant loci (rs199512, rs45608532, rs13017199, and rs3094128), three of which are in—or expression quantitative trait loci of—genes encoding proteins involved in brain development and patterning. These included microtubule-related *MAP2* and *MAPT*, as well as *WNT3* and *MICB*, all implicated in the pathogenesis of diseases such as Parkinson’s, Alzheimer’s and schizophrenia. In particular, with rs199512, we identified a common genetic influence on handedness, psychiatric phenotypes, Parkinson’s disease, and the integrity of white matter tracts connecting the same language-related regions identified in the handedness–imaging analysis. This study has identified in the general population genome-wide significant loci for human handedness in, and expression quantitative trait loci of, genes associated with brain development, microtubules and patterning. We suggest that these genetic variants contribute to neurodevelopmental lateralization of brain organization, which in turn influences both the handedness phenotype and the predisposition to develop certain neurological and psychiatric diseases.

## Teaser

The brain signature and genetic basis of handedness are unclear. Wiberg *et al.* show that left-handers have higher functional connectivity between language networks, and identify four genomic regions associated with handedness. Variants within these regions appear, by influencing brain architecture, to predispose both to left-handedness and to certain neuropsychiatric diseases.

## Introduction

One of the most remarkable features of human motor control is that ∼90% of the population has had a preference for using their right hand over the left since at least the Paleolithic period (Faurie and Raymond, 2004), and this skew in distribution of handedness is a uniquely human trait. It is widely believed that the lateralization of language in the left hemisphere accounts for the evolution of right-handedness in the majority of humans (Corballis, 2003). There are well-established associations between left-handedness and several neurodevelopmental disorders (Brandler and Paracchini, 2014); in particular, a meta-analysis of 50 studies concluded that non-right-handedness was significantly more common in participants with schizophrenia [odd ratio (OR) = 1.55, 95% confidence interval (CI) 1.25–1.93] (Hirnstein and Hugdahl, 2014).

Neuroanatomical studies of human handedness have been equivocal, most likely owing to small- to medium-sized study populations (Hatta, 2007; Guadalupe *et al.*, 2014). While studies dedicated to one specific cortical feature, such as the shape and depth of the central sulcus (Amunts *et al.*, 1996; Sun *et al.*, 2012), or the gyrification pattern of Heschl’s gyrus (Marie *et al.*, 2015), have shown differences in left-handers, no significant cortical area correlates of handedness were found in the largest study sample so far (106 left-handed subjects, 1960 right-handed subjects) (Guadalupe *et al.*, 2014). Functional imaging in the motor cortex has largely been inconclusive (Hatta, 2007). Conversely, differences in the lateralization pattern of language function have been consistently observed, with left-handers showing more bilateral or right-hemispheric language activation (Tzourio *et al.*, 1998; Pujol *et al.*, 1999; Knecht, 2002; Joliot *et al.*, 2016).

Another unresolved issue is whether such a population bias in handedness is under genetic influence. While left-handedness runs in families (Medland *et al.*, 2009), and concordance of handedness is greater in monozygotic twins than dizygotic twins, with an estimated heritability of 25% (Medland *et al.*, 2006), significantly associated loci for human handedness in the general population have thus far remained elusive (Eriksson *et al.*, 2010).

UK Biobank is a prospective cohort study of ∼500 000 participants who have allowed linkage of their physical data, including genetics, with their medical records, lifestyle questionnaires, and cognitive measures. An imaging extension includes six distinct modalities covering structural, diffusion and functional imaging of the brain, with an automatic pipeline generating thousands of image-derived phenotypes (IDPs), which are distinct individual measures that can be used for correlation with other phenotypes, or for genetic analysis (Miller *et al.*, 2016; Alfaro-Almagro *et al.*, 2018; Elliott *et al.*, 2018).

Using imaging, genotype and handedness data from UK Biobank, we aimed to discover correlations between: (i) handedness phenotype and IDPs; (ii) genotype and handedness; and (iii) handedness-related genotypes and IDPs. Supplementary Fig. 1 summarizes the key findings from the three arms of this study.

## Materials and methods

### Imaging–handedness analysis

All UK Biobank imaging data were processed following pipelines designed to create a set of IDPs that summarizes the information across all brain structural and functional modalities (Miller *et al.*, 2016; Alfaro-Almagro *et al.*, 2018). These pipelines were developed mostly using FSL tools (Jenkinson *et al.*, 2012), using well-known, validated and robust approaches for each set of IDPs: FSL-VBM (voxel-based morphometry) (Good *et al.*, 2001; Douaud *et al.*, 2007) and FreeSurfer (Dale *et al.*, 1999; Fischl *et al.*, 1999) for regional grey matter volumetric, thickness and area measures, tract-based spatial statistics (TBSS) (Smith *et al.*, 2006) and Autoptx (De Groot *et al.*, 2013) for regional diffusion measures, or FSLnets (Smith *et al.*, 2013) for functional connectivity (see list of URLs provided in the Supplementary material). The description of this recently expanded set of 3144 IDPs has been recently published (Elliott *et al.*, 2018), including full details of their estimated heritability that is summarized in Supplementary Table 1. Briefly, these comprised mainly regional volumetric, area and thickness measures; subcortical measures of MRI modalities sensitive to e.g. venous vasculature or microbleeds and white matter lesions, white matter tract measures of physical connection (‘structural connectivity’) between brain regions using diffusion indices, and measures of spontaneous temporal synchronization (‘functional connectivity’) between pairs of brain regions. IDPs were quantile normalized to ensure normality, and confounds, including age, sex, interaction between age and sex, head size, as well as various variables related to the MRI acquisition protocol, were included in the model.

We tested the effects of self-reported handedness directly in UK Biobank (Data Field 1707), and results were Bonferroni-corrected for multiple comparisons across all 3144 IDPs. This analysis was performed on the subset of imaged UK Biobank participants that had been preprocessed using the pipelines mentioned above (second release: ∼9000), by directly contrasting 721 left-handers with 6685 right-handers (all analyses excluded ambidextrous subjects).

### Genotype–handedness analysis

After performing quality control of UK Biobank genotype (including restricting samples to individuals of white British ancestry), we undertook three genome-wide association analyses across 547 011 genotyped single nucleotide polymorphisms (SNPs) and ∼11 million imputed SNPs, with genetic sex and genotyping platform used as covariates: (i) left-handers (*n = *38 332) versus right-handers (*n = *356 567); (ii) non-right handers [left-handers + ambidextrous (*n = *44 631)] versus right-handers (*n = *356 567); and (iii) left-handers (*n = *38 332) versus non-left-handers [right-handers + ambidextrous (*n = *362 866)].

### Enrichment and correlation analyses with clinical phenotypes of handedness-associated SNPs

To identify the biological pathways and gene ontologies enriched in this genome-wide association study (GWAS), we performed a SNP-based enrichment analysis and a gene-based analysis. We then analysed the average expression of the mapped genes across 53 tissue types, to gain insight into the relative tissue expressions of these mapped genes in a broad range of tissues. We also performed linkage disequilibrium (LD) score regression on summary-level statistics for the left- versus right-handers GWAS to estimate the SNP heritability, and to estimate the genetic correlation between handedness and various neurological and psychiatric diseases from publicly available summary-level GWAS data. Finally, we looked for correlations with clinical phenotypes collected directly from the entire UK Biobank population (corrected for multiple comparisons across phenotypes, *n = *1345 and loci, *n = *4).

### Genotype–imaging analysis

For the genotype–imaging study, we used BGENIE v1.2 to carry out GWA analyses of the significant loci for handedness against each of the IDPs [see URLs in the Supplementary material for BGENIE, and the Oxford Brain Imaging Genetics (BIG) web browser, which allows users to browse associations by SNP, gene or phenotype]. Results were considered significant after Bonferroni correction for multiple comparisons across all IDPs (*n = *3144) and loci (*n = *4).

As all the participants’ diffusion images are non-linearly registered to a common space (Alfaro-Almagro *et al.*, 2018), we were then able to carry out a voxel-by-voxel analysis of the most consistent result identified with our IDPs using regression against the count of the non-reference allele (0, 1 and 2). This was performed to display the full spatial extent of the relevant variants’ effects, and to investigate whether any apparent lateralization of the IDP results might be due to slight differences in significance (relative to threshold). Results were considered significant after a conservative Bonferroni correction for multiple comparisons across space (number of voxels in the image mask used to carry out the statistical analyses).

These significant voxelwise results in the white matter were then subsequently used as starting points (seed masks) for the virtual reconstruction and identification of the tracts to which they belong. For this, we ran the probabilistic tractography tool from FSL (probtrackx) with default settings (Behrens *et al.*, 2003) on 100 randomly chosen imaged UK Biobank participants.

Further details of the methodology and results are given in the Supplementary material.

### Data availability

Summary statistics from the GWAS have been deposited to ORA-Data, https://doi.org/10.5287/bodleian:zgdzgdoq1.

## Results

### Handedness and imaging: left-handers have stronger functional connectivity between right and left language networks

Directly comparing all 3144 IDPs between the brain-scanned UK Biobank participants (721 left-handers and 6685 right-handers) yielded numerous significant results, all but one using resting-state functional MRI measures (Supplementary Table 2).

**Figure 1:**
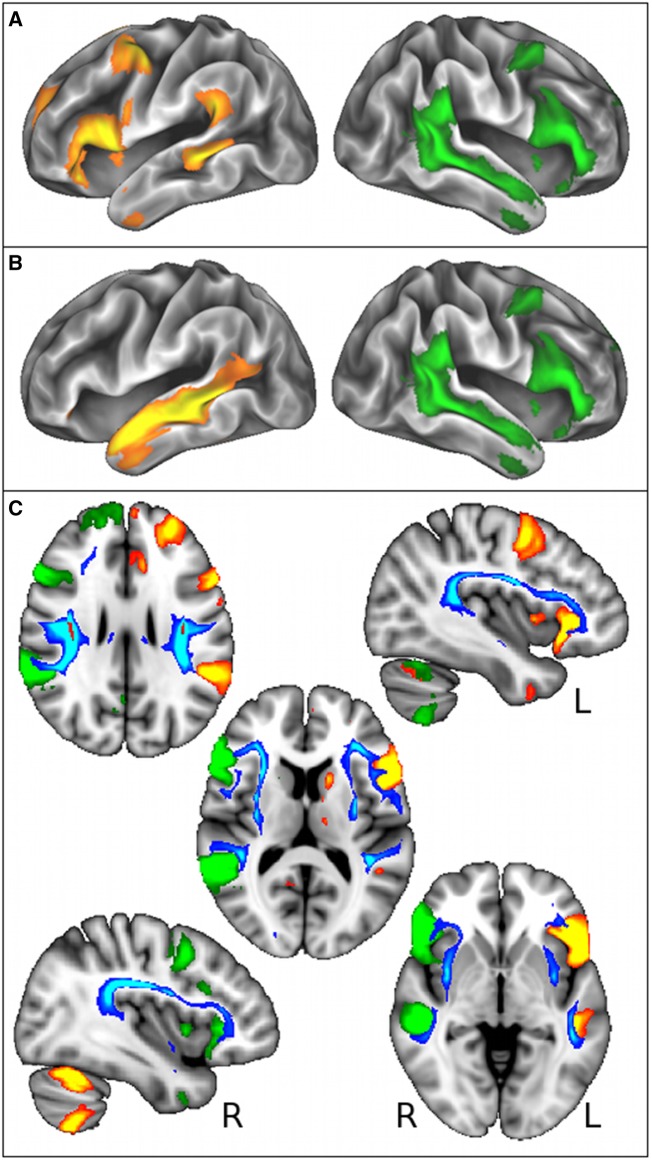
**Language-related grey matter regions functionally involved with self-reported handedness are connected by white matter tracts associated with rs199512.** (**A** and **B**) Left-handedness was most strongly associated with an increase in functional connectivity (temporal correlation) between right homologous language functional network (in green, encompassing Broca’s areas, the planum temporale and superior temporal sulcus, Z > 5), and a split of the left language functional network (in red-yellow, Broca’s areas and planum temporale shown in **A**, superior temporal sulcus shown in **B**, Z > 5). These language-related functional networks are overlaid on the cortical surface. (**C**) Voxelwise effects in white matter associated with rs199512 (in red, *P* < 3.6 × 10^−7^) were used as seeds for probabilistic tractography, which reconstructed the arcuate and superior longitudinal fasciculus (III) (in blue-light blue, thresholded for better visualization at 250 samples). Results are overlaid on the MNI T_1_-weighted template (axial views: *z* = 27, 12,−3 mm; sagittal views: *x* = −39, 39 mm). These white matter tracts clearly link the grey matter areas present in lateralized right- and left-sided language functional networks (in green and red-yellow, respectively, also shown in **A** and **B**).

The top 10 associations were all measures of functional connectivity between pairs of resting-state networks (‘edges’), the most prevalent being the homologue of the language network in the right hemisphere, encompassing Broca’s area (BA44 and 45), regions around the superior temporal sulcus, as well as premotor and primary motor regions centred around the tongue and mouth. Overall, these functional connectivity results showed, in left-handers, (i) a stronger connectivity between right and left (homologous) language networks (Fig. 1A and B); and (ii) a weaker connectivity between the right homologous language network and the default-mode network (DMN) and salience network (Supplementary Table 2A, ‘stronger connectivity’ corresponds to higher absolute values of partial correlation between the time courses of the two resting-state networks involved).

### Handedness and genetics: three of four genome-wide significant loci involve brain development and patterning genes

**Figure 2:**
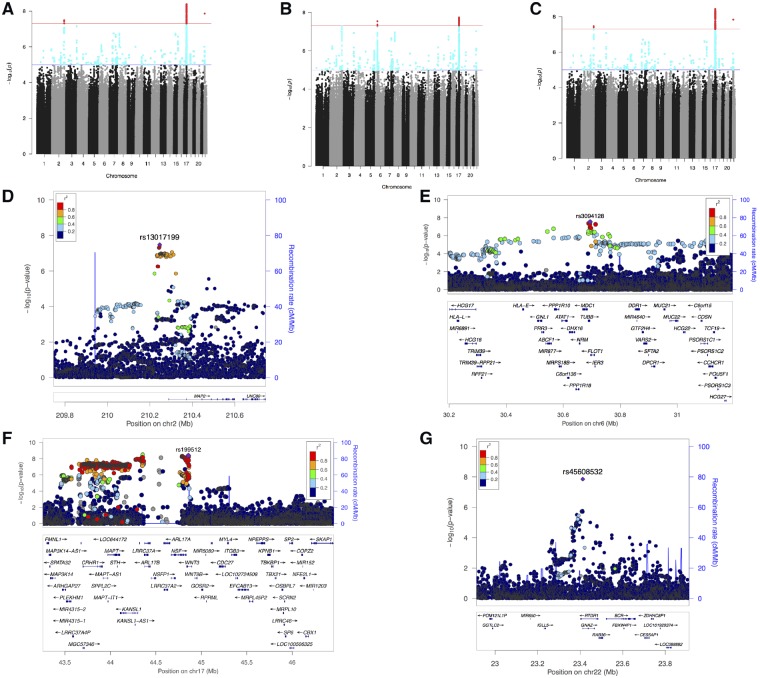
**Summary of GWAS of handedness.** (**A**–**C**) Manhattan plots showing −log10 *P*-values for SNP associations in GWAS of: (**A**) right-handers versus left-handers, (**B**) right-handers versus non-right-handers, and (**C**) left-handers versus non-left handers. Variants coloured in red have genome-wide significant associations (*P = *5 × 10^−8^). (**D**–**F**) Regional association plots of the associated SNPs: (**D**) rs13017199 at 2q34, (**E**) rs3094128 at 6p21.33, (**F**) rs199512 at 17q21.31 (with a wider window to show the entirety of the *MAPT* region), and (**G**) rs45608532 at 22q11.22, where the low linkage disequilibrium relationship between the SNPs is consistent with what has previously been reported in this region on chromosome 22 (Dawson *et al.*, 2002). The genomic positions of the SNPs and genes is based on Human Genome build hg19.

In the genotyped UK participants, we discovered three genome-wide significant loci by comparing 38 322 left-handers versus 356 567 right-handers: rs199512, 17q21.31, *P = *4.1 × 10^−9^; rs45608532, 22q11.22, *P = *1.4 × 10^−8^; rs13017199, 2q34, *P = *3.3 × 10^−8^. Comparing non-right-handers (left-handers + ambidextrous individuals) versus right-handers uncovered one further locus: rs3094128 at 6p21.33, *P = *2.9 × 10^−8^, and replicated the association at rs199512. Comparing left-handers versus non-left-handers, did not yield any new associated loci, but replicated the three loci from left- versus right-handers (Table 1 and Fig. 2). We calculated the heritability (h^2^) of handedness explained by all the SNPs in the left- versus right-handers GWAS to be 0.0121 (standard error 0.0014).

**Table 1:** Loci significantly associated with left-handedness

**Chromosome**	**Position** **^a^**	**Index SNP**	**Effect allele**	**EAF_L_** **^b^**	**EAF_R_** **^c^**	**INFO** **^d^**	**OR (95% CI)**	***P***	**Candidate genes** **^e^**
2	210246064	rs13017199	G	0.368	0.361	0.997	1.04 (1.03–1.06)	3.3 × 10^−8^	*MAP2*
6^f^	30694374	rs3094128	C	0.211	0.220	1.000	0.95 (0.94–0.97)	2.9 × 10^−8^	*TUBB*, *MICB*, *FLOT1*
17	44857352	rs199512	C	0.791	0.782	1.000	1.06 (1.04–1.07)	4.1 × 10^−9^	*WNT3*, *MAPT*, *MAPT-AS1*
22	23412190	rs45608532	A	0.075	0.069	0.942	1.09 (1.06–1.12)	1.4 × 10^−8^	*RTDR1*, *GNAZ*

^a^Based on NCBI Genome Build 37 (hg19).

^b^The effect allele frequency in left-handers (or left-handers + ambidextrous individuals in the case of rs3094128 on chromosome 6).

^c^The effect allele frequency in right-handers.

^d^The SNP INFO score for imputed SNPs.

^e^Based on eQTL data (Supplementary Table 3), positional gene mapping in FUMA (Supplementary Fig. 2), and biological plausibility.

^f^Only significant in non-right handers (i.e. left-handers + ambidextrous) versus right-handers GWAS.

The locus rs13017199 is ∼40 kb upstream of, and an expression quantitative trait locus (eQTL) of *MAP2* (microtubule-associated protein 2), rs3094128 is ∼1.2 kb downstream of *TUBB* (tubulin beta class 1), ∼1 kb upstream of *FLOT1*, and an eQTL of *MICB.* rs199512, which lies in an intron of *WNT3*, is an eQTL of *MAPT* and *MAPT-AS1*, and is located within a large LD block within a common inversion polymorphism referred to as the *MAPT* (microtubule associated protein tau) locus (Table 1 and Supplementary Table 3).

### Handedness loci are enriched for neuronal development and neurodegenerative phenotypes

The gene-based analysis demonstrated that the four gene sets and gene ontology terms with the most overlapped genes pertained to neuronal morphogenesis, differentiation, migration and gliogenesis (Supplementary Table 4). The SNP-based enrichment analysis on all nominally significant SNPs (*P* ≤ 5 × 10^−5^) in the same GWAS showed that the top two enrichments were for Parkinson’s disease (*P = *2.6 × 10^−19^) and neurodegenerative disease (*P = *2.8 × 10^−12^); of the top 10 enrichments, eight were for neurological disorder phenotypes (Supplementary Table 5). Consistent with this enrichment, positional gene mapping of the left- versus right-handers GWAS summary statistics revealed a set of genes that are highly expressed in several brain tissues (Supplementary Fig. 2).

### Genetics of handedness correlate with psychiatric phenotypes and Parkinson’s disease

First, we performed LD score regression to examine correlations between our GWAS of left-handedness and neurodegenerative and psychiatric phenotypes obtained from publicly available GWAS datasets, on the LDHub interface. Our most statistically significant correlations were with schizophrenia (r_g_ = 0.1324, *P = *0.0021), Parkinson’s disease (r_g_ = −0.2379, *P = *0.0071), and at a trend level with anorexia nervosa (r_g_ = 0.1504, *P = *0.011) and bipolar disorder (r_g_ = 0.1548, *P = *0.025) (Supplementary Table 6). Then, by investigating correlations with clinical phenotypes collected directly from the UK Biobank participants, we found significant positive associations between our handedness-associated loci and numerous mental health phenotypes (rs199512 and rs3094128), as well as a negative correlation between the allele predisposing to left-handedness at rs199512 and having a family history of Parkinson’s on the maternal side (and at trend level on the paternal side: beta = 0.0014, *P*_uncorrected_ = 0.004) (Table 2).

**Table 2:** Correlation with clinical phenotypes collected from the UK Biobank participants

**Locus**	**Category**	**UK Biobank phenotype**	**Beta**	**Direction** **^a^**	**Uncorrected *P***
rs199512	**Mental health**	**Neuroticism score**	**−0.086**	**+**	**3.70 × 10^−16^**
	**Mental health**	**Sensitivity / hurt feelings**	**−0.013**	**+**	**5.10 × 10^−18^**
	**Mental health**	**Mood swings**	**−0.011**	**+**	**1.20 × 10^−13^**
	**Mental health**	**Frequency of tenseness / restlessness in last 2 weeks**	**−0.011**	**+**	**4.80 × 10^−10^**
	**Illnesses**	**Pain type(s) experienced in last month: headache**	**−0.0089**	**+**	**4.60 × 10^−14^**
	**Mental health**	**Fed-up feelings**	**−0.0087**	**+**	**2.90 × 10^−9^**
	**Mental health**	**Irritability**	**−0.0081**	**+**	**2.00 × 10^−9^**
	**Mental health**	**Miserableness**	**−0.0077**	**+**	**1.20 × 10^−7^**
	**Mental health**	**Worrier / anxious feelings**	**−0.007**	**+**	**1.60 × 10^−6^**
	**Mental health**	**Nervous feelings**	**−0.0068**	**+**	**8.80 × 10^−8^**
	Illnesses	Mouth/teeth dental problems: mouth ulcers	−0.0054	+	1.40 × 10^−9^
	**Family illnesses**	**Illnesses of mother: Parkinson’s disease**	**0.0018**	**−**	**1.90 × 10^−6^**
	Non-cancer illness	Non-cancer illness code, self-reported: helicobacter pylori	0.0007	−	3.70 × 10^−6^
rs3094128	**Mental health**	**Seen doctor (GP) for nerves, anxiety, tension or depression**	**−0.0094**	**+**	**1.00 × 10^−11^**
	Non-cancer illness	Non-cancer illness code, self-reported: asthma	0.0077	−	4.70 × 10^−16^
	Illnesses	Diagnosed by doctor: Asthma	0.0076	−	9.50 × 10^−16^
	Illnesses	Hearing difficulty/problems with background noise	−0.0072	+	5.50 × 10^−7^
	Illnesses	Mouth/teeth dental problems: dentures	0.0066	−	2.10 × 10^−9^
	Non-cancer illness	Non-cancer illness code, self-reported: malabsorption/coeliac disease	0.0055	−	9.8 × 10^−182^
	**Mental health**	**Tense / ‘highly strung’**	**−0.0052**	**+**	**4.60 × 10^−6^**
	**Mental health**	**Seen a psychiatrist for nerves, anxiety, tension or depression**	**−0.005**	**+**	**6.90 × 10^−8^**
	Illnesses	Eye problems/disorders: diabetes-related eye disease	0.0034	−	1.80 × 10^−6^
	Non-cancer illness	Non-cancer illness code, self-reported: hypothyroidism/myxoedema	0.0033	−	9.60 × 10^−8^
	Non-cancer illness	Non-cancer illness code, self-reported: hyperthyroidism/thyrotoxicosis	0.0029	−	3.40 × 10^−29^
	Illnesses	Diabetes diagnosed by doctor	0.0029	−	5.10 × 10^−6^
	Illnesses	Doctor diagnosed sarcoidosis	0.0027	−	6.50 × 10^−13^
	ICD-10 codes	Diagnoses: main ICD10: R31 Unspecified haematuria	−0.0027	+	2.50 × 10^−10^
	ICD-10 codes	Diagnoses: main ICD10: K90 Intestinal malabsorption	0.0022	−	8.80 × 10^−68^
	Non-cancer illness	Non-cancer illness code, self-reported: psoriasis	−0.0017	+	2.90 × 10^−8^
	Non-cancer illness	Non-cancer illness code, self-reported: enlarged prostate	−0.0016	+	1.20 × 10^−6^
	ICD-10 codes	Diagnoses: main ICD10: N40 Hyperplasia of prostate	−0.0013	+	3.30 × 10^−6^
	Non-cancer illness	Non-cancer illness code, self-reported: ankylosing spondylitis	−0.0008	+	2.70 × 10^−7^
	Non-cancer illness	Non-cancer illness code, self-reported: sarcoidosis	0.0008	−	4.90 × 10^−10^
	Non-cancer illness	Non-cancer illness code, self-reported: systemic lupus erythematosis	0.0005	−	2.90 × 10^−7^
	ICD-10 codes	Diagnoses: main ICD10: E10 Insulin-dependent diabetes mellitus	0.00048	−	4.40 × 10^−6^
	Non-cancer illness	Non-cancer illness code, self-reported: type 1 diabetes	0.00039	−	5.80 × 10^−6^

Two loci associated with left-handedness (rs199512 and rs3094128) were also significantly associated with numerous mental health variables and with familial history of Parkinson’s disease in the genotyped UK Biobank participants. Only results surviving correction for multiple comparisons across loci (*n* = 4) and across clinical phenotypes (*n* = 1345, Supplementary material) are presented, and these are ranked by effect size. Clinical phenotypes directly related to neurological or mental health symptoms are highlighted in bold.

^a^Direction refers of the correlation between the phenotype in question and the allele that predisposes to non-right-handedness for rs199512 and rs3094128. A positive value indicates that the allele predisposing to non-right-handedness is positively correlated with the phenotype.

### Genetics of handedness and imaging: rs199512 is associated with structural connectivity between language areas

Consistent with rs199512 being in a gene coding for—and an eQTL of—proteins involved in brain development and axonal guidance (*WNT3*, *MAPT*, *MAPT-AS1*), this SNP yielded many highly significant associations with measures of white matter structural connectivity (diffusion imaging IDPs) (Supplementary Table 7). In particular, those differences were revealed most strongly in tracts linking Broca’s and temporoparietal junction areas (arcuate/superior longitudinal fasciculus III), i.e. specifically the same brain regions found differentially functionally-connected in our direct handedness and imaging analysis (Fig. 1C).

## Discussion

Through our top SNP associated with handedness, rs199512, we have identified a common genetic influence on handedness, Parkinson’s disease and many mental health phenotypes (such as neuroticism, or mood swings, Table 2) and the integrity of the arcuate and superior longitudinal fasciculus (III) fasciculus.

These language-related tracts have been consistently associated with schizophrenia and auditory hallucinations (Hubl *et al.*, 2004). The lack of lateralization in our white matter results might be surprising at first, but seems to be consistent with a recent study that failed to find any significant associations of handedness with grey matter asymmetries (Kong *et al.*, 2018). We also found no significant difference in the IDP of grey matter structure (grey matter volume, as well as cortical thickness and area) between left- and right-handers, including that of any asymmetry, although we did not assess specifically the shape/depth of the central sulcus, or the gyrification pattern of the Heschl’s gyrus. Of note, however, the white matter tracts associated with rs199512 link grey matter regions known to show the strongest asymmetries from an early developmental stage (Dubois *et al.*, 2010).

Remarkably, all of the grey matter regions connected by these language-related white matter tracts specifically make up the functional (homologous) language networks that differ between left- and right-handers (Fig. 1). Our findings of a stronger functional connectivity—in this case higher positive functional connectivity—between right and left (homologous) language networks in left-handers is consistent with imaging studies that have showed more symmetrical functional activations in language comprehension and language generation in left-handers (Tzourio *et al.*, 1998; Pujol *et al.*, 1999; Knecht, 2002; Joliot *et al.*, 2016). One of the early studies demonstrated a linear relationship between the rate of right-lateralization of language dominance and the degree of left-handedness (Knecht, 2002), while the largest functional study to date (153 left-handers, 137 right-handers) showed a strong atypical pattern of lateralization for language production in 7% of left-handers, but in no right-handers (Joliot *et al.*, 2016). Additional evidence for the stronger involvement of right language-related brain regions in left-handers could be seen in our results with a weaker suppression of the DMN influence (Anticevic *et al.*, 2012) on the right language functional network. Except for one single ‘edge’ in the lower dimension decomposition (Supplementary Table 2B: ICA25 edge 50, r = 0.04, just above significance level at 10^−6^), we found no effect of handedness on motor networks. While there might be confounds to such functional connectivity measures (Friston, 2011; Duff *et al.*, 2018), we found no association in particular with physiological measures of heart rate and blood pressure (systolic and diastolic) for the two topmost significant edges (visualized in Fig. 1A and B).

As the effect of polymorphisms related to handedness could be seen specifically in language-related tracts connecting the brain regions of the language networks, our functional connectivity findings may thus be the hallmarks in the adult brain of some very early genetically-guided events happening in the white matter cytoskeleton during development. Such genetic effects in the human white matter probably mirror similar, very early cytoskeletal processes observed in the development of chirality in gastropods and amphibians (Davison *et al.*, 2016). It is thus perhaps unsurprising that, in total, three of the four loci correlating with handedness in our GWAS are indeed associated with genes strongly involved in brain development and patterning (*MAP2*, *TUBB*/*MICB*, *WNT3*/*MAPT*). In particular, microtubules (*MAP2*, *TUBB*, *MAPT*)—as integral components of the neuronal cytoskeleton—play a key role in neuronal morphogenesis and migration. WNT3 has also been shown to act as an axon guidance molecule and, strikingly, as a gradient for retinotopic mapping along the medial-lateral axis (Schmitt *et al.*, 2006). Of note, rs3094128 is an eQTL of *MICB*, which is crucial to brain development and plasticity and may mediate both genetic and environmental involvements in schizophrenia (McAllister, 2014).

There is a plethora of literature demonstrating a preponderance of left-handedness in an array of psychiatric disorders, including meta-analyses in schizophrenia (Hirnstein and Hugdahl, 2014), supporting the view that there is a genetic link between handedness, brain lateralization and schizophrenia (Berlim *et al.*, 2003; Francks *et al.*, 2007). In line with this, we found a statistically significant positive correlation between left-handedness and schizophrenia using LD score regression (Supplementary Table 6).

Perhaps the best-known pathological associations of *MAPT* are Parkinson’s and Alzheimer’s diseases, with evidence for genetic overlap between these two neurodegenerative disorders within this extended *MAPT* region (Desikan *et al.*, 2015). Several polymorphisms in and around *MAPT* have been discovered in GWAS of Parkinson’s disease (Satake *et al.*, 2009). Those SNPs likely account for the genetic enrichment observed between handedness and Parkinson’s disease in both our LD score regression and SNP-based enrichment analyses. We also identified a strong negative relationship between the allele predisposing to left-handedness at rs199512, which is an eQTL of *MAPT* and *MAPT-AS1* (Supplementary Table 3), and a diagnosis of Parkinson’s disease for the mother of the UK Biobank participants (Table 2). This association—only seen in the parent of the participants—is probably a reflection of the relatively young recruitment age in the UK Biobank (40–69), meaning that only a few genetically-susceptible individuals will have developed the disease themselves at that age. The negative association between the left-handedness predisposing allele and a maternal family history of Parkinson’s disease is consistent with the LD score regression analyses, where Parkinson’s disease also showed a negative correlation with left-handedness, in contrast to the majority of phenotypes examined. Notably, rs199512 is also in an intron of *WNT3*, which has itself been implicated in Parkinson’s disease (Simón-Sánchez *et al.*, 2009).

Findings from previous GWAS and neuroimaging studies of human handedness have been equivocal, with a few exceptions (Hatta, 2007; Scerri *et al.*, 2011; Brandler *et al.*, 2013), most likely owing to small- to medium-sized study populations (Guadalupe *et al.*, 2014). The considerable size of the UK Biobank cohort and imaging sub-cohort (∼400 00 and ∼9000, respectively) has allowed us to discover novel loci and correlations between handedness and imaging phenotypes. The relatively modest effect sizes of the associated variants, and the heritability estimate of handedness explained by all SNPs in the left- versus right-handers GWAS (0.012), are consistent with a polygenic model of inheritance incorporating many variants of very low effect size. Similarly, the strongest effect of handedness in the brain explained about 1.4% of the variance seen in functional connectivity between the two language-related networks, in contrast with the larger—albeit still modest—effects seen in studies using dedicated language task-functional MRI or functional transcranial Doppler sonography (Tzourio *et al.*, 1998; Pujol *et al.*, 1999; Groen *et al.*, 2013; Joliot *et al.*, 2016).

This is the first study to identify in the general population genome-wide significant loci for human handedness in, and eQTL of, genes associated with brain development, microtubules and patterning. While replication in a large, well-powered independent cohort is needed to confirm these associations, it is striking that the associated loci are also strongly positively correlated with schizophrenia and negatively correlated with Parkinson’s disease. In particular, our most significant SNP, rs199512, was not only directly associated with mental health phenotypes and familial history of Parkinson’s disease in the UK Biobank participants, but also with structural connectivity measures in white matter tracts connecting language-related brain areas. Thus, this locus has biological plausibility in contributing to differences in neurodevelopmental connectivity of language areas. The lateralization of brain language function was strongly related to handedness; whether increased bilateral language function gives left-handers a cognitive advantage at verbal tasks remains to be investigated separately in a large dataset offering both well-characterized verbal cognition testing (not available in UK Biobank) as well as FSLnets-like functional connectivity measures, such as the Human Connectome Project (Anticevic *et al.*, 2012; Van Essen *et al.*, 2013; Somers *et al.*, 2015). This study thus represents an important advance in our understanding of human handedness and offers mechanistic insights into the observed correlations between chirality and microtubules in the brain, and suggests an overlap of genetic architecture between handedness and certain neurodegenerative and psychiatric phenotypes.

## Supplementary Material

awz257_Supplementary_Data

## Abbreviations

eQTL: expression quantitative trait loci
GWAS: genome-wide association study
IDP: image-derived phenotype
SNP: single nucleotide polymorphism

## References

[awz257-B1] Alfaro-Almagro F, Jenkinson M, Bangerter NK, Andersson JLR, Griffanti L, Douaud G et al Image processing and quality control for the first 10,000 brain imaging datasets from UK Biobank. Neuroimage 2018; 166: 400–24. 29079522 10.1016/j.neuroimage.2017.10.034 PMC5770339

[awz257-B2] Amunts K, Schlaug G, Schleicher A, Steinmetz H, Dabringhaus A, Roland PE et al Asymmetry in the human motor cortex and handedness. Neuroimage 1996; 4: 216–222. 9345512 10.1006/nimg.1996.0073

[awz257-B3] Anticevic A, Cole MW, Murray JD, Corlett PR, Wang XJ, Krystal JH The role of default network deactivation in cognition and disease. Trends Cogn Sci 2012. 10.1016/j.tics.2012.10.008 PMC3501603 23142417

[awz257-B4] Behrens TEJ, Woolrich MW, Jenkinson M, Johansen-Berg H, Nunes RG, Clare S et al Characterization and propagation of uncertainty in diffusion-weighted MR imaging. Magn Reson Med 2003; 50: 1077–88. 14587019 10.1002/mrm.10609

[awz257-B5] Berlim MT, Mattevi BS, Belmonte-de-Abreu P, Crow TJ The etiology of schizophrenia and the origin of language: overview of a theory. Compr Psychiatry 2003; 44: 7–14. 12524630 10.1053/comp.2003.50003

[awz257-B6] Brandler WM, Morris AP, Evans DM, Scerri TS, Kemp JP, Timpson NJ et al Common variants in left/right asymmetry genes and pathways are associated with relative hand skill. PLoS Genet 2013; 9: e1003751. 24068947 10.1371/journal.pgen.1003751 PMC3772043

[awz257-B7] Brandler WM, Paracchini S The genetic relationship between handedness and neurodevelopmental disorders. Trends Mol Med 2014; 20: 84–9. 10.1016/j.molmed.2013.10.008 PMC3969300 24275328

[awz257-B8] Corballis MC From mouth to hand: gesture, speech, and the evolution of right-handedness. Behav Brain Sci 2003; 26: 199–208; discussion 208–60. 14621511 10.1017/s0140525x03000062

[awz257-B9] Dale AM, Fischl B, Sereno MI Cortical surface-based analysis: I. Segmentation and surface reconstruction. Neuroimage 1999; 9: 179–94. 9931268 10.1006/nimg.1998.0395

[awz257-B10] Davison A, McDowell GS, Holden JM, Johnson HF, Koutsovoulos GD, Liu MM et al Formin is associated with left-right asymmetry in the pond snail and the frog. Curr Biol 2016; 26: 654–60. 26923788 10.1016/j.cub.2015.12.071 PMC4791482

[awz257-B11] Dawson E, Abecasis GR, Bumpstead S, Chen Y, Hunt S, Beare DM et al A first-generation linkage disequilibrium map of human chromosome 22. Nature 2002; 418: 544–8. 12110843 10.1038/nature00864

[awz257-B12] De Groot M, Vernooij MW, Klein S, Ikram MA, Vos FM, Smith SM et al Improving alignment in tract-based spatial statistics: evaluation and optimization of image registration. Neuroimage 2013; 76: 400–11. 23523807 10.1016/j.neuroimage.2013.03.015 PMC6588540

[awz257-B13] Desikan RS, Schork AJ, Wang Y, Witoelar A, Sharma M, McEvoy LK et al Genetic overlap between Alzheimer’s disease and Parkinson’s disease at the MAPT locus. Mol Psychiatry 2015; 20: 1588–95. 25687773 10.1038/mp.2015.6 PMC4539304

[awz257-B14] Douaud G, Smith S, Jenkinson M, Behrens T, Johansen-Berg H, Vickers J et al Anatomically related grey and white matter abnormalities in adolescent-onset schizophrenia. Brain 2007; 130: 2375–86. 17698497 10.1093/brain/awm184

[awz257-B15] Dubois J, Benders M, Lazeyras F, Borradori-Tolsa C, Leuchter RHV, Mangin JF et al Structural asymmetries of perisylvian regions in the preterm newborn. Neuroimage 2010; 52: 32–42. 20362679 10.1016/j.neuroimage.2010.03.054

[awz257-B16] Duff EP, Makin T, Cottaar M, Smith SM, Woolrich MW Disambiguating brain functional connectivity. Neuroimage 2018; 173: 540–550. 29476911 10.1016/j.neuroimage.2018.01.053 PMC5929905

[awz257-B17] Elliott LT, Sharp K, Alfaro-Almagro F, Shi S, Miller KL, Douaud G et al Genome-wide association studies of brain imaging phenotypes in UK Biobank. Nature 2018; 562: 210–16. 30305740 10.1038/s41586-018-0571-7 PMC6786974

[awz257-B18] Eriksson N, Macpherson JM, Tung JY, Hon LS, Naughton B, Saxonov S et al Web-based, participant-driven studies yield novel genetic associations for common traits. PLoS Genet 2010; 6: 1–20. 10.1371/journal.pgen.1000993 PMC2891811 20585627

[awz257-B19] Faurie C, Raymond M Handedness frequency over more than ten thousand years. Proc R Soc B Biol Sci 2004; 271: S43–5. 10.1098/rsbl.2003.0092 PMC1809976 15101415

[awz257-B20] Fischl B, Sereno MI, Dale AM Cortical surface-based analysis: II. Inflation, flattening, and a surface-based coordinate system. Neuroimage 1999; 9: 195–207. 9931269 10.1006/nimg.1998.0396

[awz257-B21] Francks C, Maegawa S, Laurén J, Abrahams BS, Velayos-Baeza A, Medland SE et al LRRTM1 on chromosome 2p12 is a maternally suppressed gene that is associated paternally with handedness and schizophrenia. Mol Psychiatry 2007; 12: 1129–39. 17667961 10.1038/sj.mp.4002053 PMC2990633

[awz257-B22] Friston KJ Functional and effective connectivity: a review. Brain Connect 2011; 1: 13–36. 22432952 10.1089/brain.2011.0008

[awz257-B23] Good CD, Johnsrude IS, Ashburner J, Henson RNA, Friston KJ, Frackowiak RSJ A voxel-based morphometric study of ageing in 465 normal adult human brains. Neuroimage 2001; 14: 21–36. 11525331 10.1006/nimg.2001.0786

[awz257-B24] Groen MA, Whitehouse AJO, Badcock NA, Bishop DVM Associations between handedness and cerebral lateralisation for language: a comparison of three measures in children. PLoS One 2013. 10.1371/journal.pone.0064876 PMC3667815 23738006

[awz257-B25] Guadalupe T, Willems RM, Zwiers MP, Vasquez AA, Hoogman M, Hagoort P et al Differences in cerebral cortical anatomy of left-and right-handers. Front Psychol 2014; 5: 261. 24734025 10.3389/fpsyg.2014.00261 PMC3975119

[awz257-B26] Hatta T Handedness and the brain: a review of brain-imaging techniques. Magn Reson Med Sci 2007; 6: 99–112. 17690540 10.2463/mrms.6.99

[awz257-B27] Hirnstein M, Hugdahl K Excess of non-right-handedness in schizophrenia: meta-analysis of gender effects and potential biases in handedness assessment. Br J Psychiatry 2014; 205: 260–7. 25274314 10.1192/bjp.bp.113.137349

[awz257-B28] Hubl D, Koenig T, Strik W, Federspiel A, Kreis R, Boesch C et al Pathways that make voices: white matter changes in auditory hallucinations. Arch Gen Psychiatry 2004; 61: 658–68. 15237078 10.1001/archpsyc.61.7.658

[awz257-B29] Jenkinson M, Beckmann CF, Behrens TEJ, Woolrich MW, Smith SM FSL. Neuroimage 2012; 62: 782–90. 21979382 10.1016/j.neuroimage.2011.09.015

[awz257-B30] Joliot M, Tzourio-Mazoyer N, Mazoyer B Intra-hemispheric intrinsic connectivity asymmetry and its relationships with handedness and language Lateralization. Neuropsychologia 2016; 93: 437–47. 26988116 10.1016/j.neuropsychologia.2016.03.013

[awz257-B31] Knecht S Handedness and hemispheric language dominance in healthy humans. Brain 2002; 123: 2512–18. 10.1093/brain/123.12.2512 11099452

[awz257-B32] Kong X-Z, Mathias SR, Guadalupe T, Enigma Consortium, Glahn DC, Franke B et al Mapping cortical brain asymmetry in 17,141 healthy individuals worldwide via the ENIGMA Consortium. Proc Natl Acad Sci 2018; 115: E5154–63. 29764998 10.1073/pnas.1718418115 PMC5984496

[awz257-B33] Marie D, Jobard G, Crivello F, Perchey G, Petit L, Mellet E et al Descriptive anatomy of Heschl’s gyri in 430 healthy volunteers, including 198 left-handers. Brain Struct Funct 2015; 220: 729–43. 10.1007/s00429-013-0680-x PMC4341020 24310352

[awz257-B34] McAllister AK Major histocompatibility complex i in brain development and schizophrenia. Biol Psychiatry 2014; 75: 262–8. 24199663 10.1016/j.biopsych.2013.10.003 PMC4354937

[awz257-B35] Medland SE, Duffy DL, Wright MJ, Geffen GM, Hay DA, Levy F et al Genetic influences on handedness: data from 25,732 Australian and Dutch twin families. Neuropsychologia 2009; 47: 330–7. 18824185 10.1016/j.neuropsychologia.2008.09.005 PMC2755095

[awz257-B36] Medland SE, Duffy DL, Wright MJ, Geffen GM, Martin NG Handedness in twins: joint analysis of data from 35 samples. Twin Res Hum Genet 2006; 9: 46–53. 16611467 10.1375/183242706776402885

[awz257-B37] Miller KL, Alfaro-Almagro F, Bangerter NK, Thomas DL, Yacoub E, Xu J et al Multimodal population brain imaging in the UK Biobank prospective epidemiological study. Nat Neurosci 2016; 19: 1523–36. 27643430 10.1038/nn.4393 PMC5086094

[awz257-B38] Pujol J, Deus J, Losilla JM, Capdevila A Cerebral lateralization of language in normal left-handed people studied by functional MRI. Neurology 1999; 52: 1038. 10102425 10.1212/wnl.52.5.1038

[awz257-B39] Satake W, Nakabayashi Y, Mizuta I, Hirota Y, Ito C, Kubo M et al Genome-wide association study identifies common variants at four loci as genetic risk factors for Parkinson’s disease. Nat Genet 2009; 41: 1303–7. 19915576 10.1038/ng.485

[awz257-B40] Scerri TS, Brandler WM, Paracchini S, Morris AP, Ring SM, Richardson AJ et al PCSK6 is associated with handedness in individuals with dyslexia. Hum Mol Genet 2011; 20: 608–14. 21051773 10.1093/hmg/ddq475 PMC3016905

[awz257-B41] Schmitt AM, Shi J, Wolf AM, Lu CC, King LA, Zou Y Wnt-Ryk signalling mediates medial-lateral retinotectal topographic mapping. Nature 2006; 439: 31–7. 16280981 10.1038/nature04334

[awz257-B42] Simón-Sánchez J, Schulte C, Bras JM, Sharma M, Gibbs JR, Berg D et al Genome-wide association study reveals genetic risk underlying Parkinson’s disease. Nat Genet 2009; 41: 1308–12. 19915575 10.1038/ng.487 PMC2787725

[awz257-B43] Smith SM, Jenkinson M, Johansen-Berg H, Rueckert D, Nichols TE, Mackay CE et al Tract-based spatial statistics: voxelwise analysis of multi-subject diffusion data. Neuroimage 2006; 31: 1487–505. 16624579 10.1016/j.neuroimage.2006.02.024

[awz257-B44] Smith SM, Vidaurre D, Beckmann CF, Glasser MF, Jenkinson M, Miller KL et al Functional connectomics from resting-state fMRI. Trends Cogn Sci 2013; 17: 666–82. 24238796 10.1016/j.tics.2013.09.016 PMC4004765

[awz257-B45] Somers M, Shields LS, Boks MP, Kahn RS, Sommer IE Cognitive benefits of right-handedness: a meta-analysis. Neurosci Biobehav Rev 2015; 51: 48–63. 25592981 10.1016/j.neubiorev.2015.01.003

[awz257-B46] Sun ZY, Klöppel S, Rivière D, Perrot M, Frackowiak R, Siebner H et al The effect of handedness on the shape of the central sulcus. Neuroimage 2012; 60: 332–39. 22227053 10.1016/j.neuroimage.2011.12.050

[awz257-B47] Tzourio N, Crivello F, Mellet E, Nkanga-Ngila B, Mazoyer B Functional anatomy of dominance for speech comprehension in left handers vs right handers. Neuroimage 1998; 8: 1–16. 9698571 10.1006/nimg.1998.0343

[awz257-B48] Van Essen DC, Smith SM, Barch DM, Behrens TEJ, Yacoub E, Ugurbil K The WU-Minn human connectome project: an overview. Neuroimage 2013; 80: 62–79. 23684880 10.1016/j.neuroimage.2013.05.041 PMC3724347

