# Supplementary material for: Handedness, language areas and neuropsychiatric diseases: insights from brain imaging and genetics
Source: Brain. 2019 Sep 5;142(10):2938–47. doi: 10.1093/brain/awz257 (PMC6763735; doi:10.1093/brain/awz257)
Supplement: awz257_Supplementary_Data [file awz257_supplementary_data.pdf]

Supplementary Material

Material and Methods

GWAS

For the three genotype:handedness association studies, we used self-reported handedness as recorded in UK Biobank Data Field 1707 – participants were invited to answer the question, "Are you right or left handed?", and could choose between “Right-handed”, “Left-handed”, and “Use both right and left hands equally” (ambidextrous). There were up to three instances when participants were asked this question; any participants who gave inconsistent responses were excluded from being classified as one of the three handedness phenotypes.

Both SNP- and sample-based quality control (QC) of UK Biobank genotype data was performed using a pipeline previously described elsewhere (Wiberg *et al.*, 2019), using PLINK v1.9 and R v3.31 (see URLs), resulting in a final dataset of 401,667 individuals and 547,011 SNPs. UK Biobank’s method of phasing and imputation of SNPs is described in detail elsewhere (Bycroft *et al.*, 2018). Briefly, phasing on the autosomes was performed using SHAPEIT3, using the 1000 Genomes Phase 3 dataset as a reference panel. For imputation, both the HRC (Haplotype Reference Consortium) reference panel and a merged UK10K / 1000 Genomes Phase 3 panel were used. This resulted in a dataset with 92,693,895 autosomal SNPs, short indels and large structural variants. Imputation files were released in the BGEN (v1.2) file format.

Within the post-QC UK Biobank dataset, there were 356,567 right-handed participants, 38,332 left-handed participants, and 6,299 ambidextrous participants.

We undertook a genome-wide association analysis across 547,011 genotyped SNPs and ~11 million imputed SNPs from the HRC panel with MAF ≥ 0.001 and Info Score ≥ 0.3, using a linear mixed non-infinitesimal model implemented in BOLT-LMM v2.3 (Loh *et al.*, 2015). For imputed SNPs, genotype dosages (rather than rounded genotypes) were used. We used a minimally adjusted model in the association analysis to avoid potential collider bias (Day *et al.*, 2016), and the following covariates were used: genetic sex and the genotyping platform (to account for array effects). While the authors of the BOLT-LMM software state that principal components can be used as covariates for the purpose of accelerating the computations (Loh *et al.*, 2018), the computational method of BOLT-LMM implicitly performs this step, so the absence of principal components as covariates does not alter the output.

We used a reference genetic map file for hg19 and a reference linkage disequilibrium (LD) score file for European-ancestry individuals included in the BOLT-LMM package in the analysis. Two covariates were used in the association study: genetic sex and the genotyping platform (to account for array effects). The LD score regression intercept (Bulik-Sullivan *et al.*, 2015*b*) of 1.0107 with an attenuation ratio of 0.1032 indicated minimal inflation when adjusted for the large sample size. Conditional analysis at each associated locus was performed by conditioning on the allelic dosage (calculated using QCTOOL v2) of the most significantly associated SNP at each locus, and we did not observe any independent associations with handedness at any of the loci.

In silico analyses of associated SNPs and regions

To identify the biological and cellular pathways underlying the association signals, we performed enrichment analyses for SNPs and genes using XGR (Fang *et al.*, 2016), a software that assists in the interpretation of GWAS statistics by incorporating ontology, annotation, and systems biology network-driven approaches. We performed a SNP-based enrichment analysis for 4,007 SNPs with a p-value suggestive of association of $p<5\times10^{-5}$ in the left- vs right-handers GWAS, excluding SNPs in LD.

For the left- vs right-handers GWAS, we used FUMA (Watanabe *et al.*, 2017) (FUnctional Mapping and Annotation of genetic associations) to map genes to the three associated loci based on physical position in the genome (positional mapping), resulting in 13 mapped genes. In order to gain insight into the relative tissue expressions of these mapped genes in a broad range of tissues, we used the GENE2FUNC tool in FUMA to look at the average expression of these genes across 53 GTEx v7 (Aguet *et al.*, 2017) tissue types.

Also for the left- vs right-handers GWAS, we performed a gene-set analysis in MAGMA (de Leeuw *et al.*, 2015) (multi-marker analysis of genomic annotation), implemented in FUMA. MAGMA uses a gene-based (rather than SNP-based) GWAS approach, whereby SNPs that are located within protein-coding genes (based on locations in NCBI build 37) are assigned a p-value describing the association found with left-handedness. The MAGMA gene-set analysis is performed for curated gene sets and GO terms obtained from MsigDB v6.1 (10655 gene sets - curated gene sets: 4738, GO terms: 5917).

Brain expression quantitative trait loci (eQTL) were obtained from GTEx (Aguet *et al.*, 2017), BRAINEAC of the UK Brain Expression Consortium (Ramasamy *et al.*, 2014), and Brain-eMeta (Qi *et al.*, 2018), and were accessed on 03/06/2019 (see URLs).

We performed LD score regression (Bulik-Sullivan *et al.*, 2015*a*; Zheng *et al.*, 2017) on summary-level statistics for the left- vs right-handers GWAS to estimate the SNP heritability, and to estimate the genetic correlation between handedness and various neurological and psychiatric diseases from publicly available summary-level GWAS data. This analysis was performed in LD Hub (see URLs). We took our summary-level GWAS data from the left- vs right-handers GWAS, keeping only the SNPs provided by LD Hub (downloaded from http://ldsc.broadinstitute.org/static/media/w_hm3.noMHC.snplist.zip). This retained 27 genome-wide significant SNPs from our GWAS. We calculated the heritability (h^2) of handedness explained by all the SNPs in the left- vs right-handers GWAS to be 0.0121 (standard error 0.0014). For the genetic correlation studies with handedness, we selected 14 phenotypes from the 'neurological diseases' and 'psychiatric diseases' categories on LDHub.

Finally, we performed a GWA regression analysis (using BGENIE v1.2) of all four significant loci for handedness against the following clinical phenotypes, made into dummy variables:

- 'Illnesses': n=140, UK Biobank code 100036,
- 'Mental health': n=41, UK Biobank code 100060,
- 'Non-cancer illness': n=341, UK Biobank code 20002,
- 'Primary cause of death': n=141, UK Biobank code 40001,
- 'Family illnesses': n=40, UK Biobank code 100034 (illnesses of mother, father, siblings),
- 'ICD10 codes': n=642, UK Biobank code 41202.

These were collected from ~337,000 UK Biobank unrelated individuals of British ancestry (see penultimate URL). Results were considered significant after a Bonferroni correction for multiple comparisons across the four loci and across all clinical phenotypes (n=1,345).

URLs

FSL-VBM https://fsl.fmrib.ox.ac.uk/fsl/fslwiki/FSLVBM

FreeSurfer http://freesurfer.net/

TBSS https://fsl.fmrib.ox.ac.uk/fsl/fslwiki/TBSS/

AutoPtx https://fsl.fmrib.ox.ac.uk/fsl/fslwiki/AutoPtx

FSLnets https://fsl.fmrib.ox.ac.uk/fsl/fslwiki/FSLNets

UK Biobank http://www.ukbiobank.ac.uk/

PLINK http://www.cog-genomics.org/plink/2.0/

R https://www.r-project.org

1000 Genomes Project http://www.1000genomes.org

QCTOOL v2 http://www.well.ox.ac.uk/~gav/qctool_v2/#overview

BGENIE https://jmarchini.org/bgenie/

XGR http://galahad.well.ox.ac.uk:3020/

FUMA http://fuma.ctglab.nl/

MAGMA https://ctg.cncr.nl/software/magma

GTEx Portal https://gtexportal.org/

BRAINEAC https://braineac.org

Brain-eMETA eQTL data https://cnsgenomics.com/software/smr/#DataResource

LD Hub http://ldsc.broadinstitute.org/ldhub/

GWAS results of ~2,000 phenotypes in the UK Biobank (Ben Neale's research group)
http://www.nealelab.is/blog/2017/7/19/rapid-gwas-of-thousands-of-phenotypes-for-337000-samples-in-the-uk-biobank

Oxford Brain Imaging Genetics (BIG) web browser **http://big.stats.ox.ac.uk/**

References

Aguet F, Ardlie KG, Cummings BB, Gelfand ET, Getz G, Hadley K, et al. Genetic effects on gene expression across human tissues. Nature 2017; 550: 204–213.

Bulik-Sullivan B, Finucane HK, Anttila V, Gusev A, Day FR, Loh PR, et al. An atlas of genetic correlations across human diseases and traits. Nat Genet 2015; 47: 1236–1241.

Bulik-Sullivan BK, Loh P-R, Finucane HK, Ripke S, Yang J, Patterson N, et al. LD Score regression distinguishes confounding from polygenicity in genome-wide association studies. Nat Genet 2015; 47: 291–295.

Bycroft C, Freeman C, Petkova D, Band G, Elliott LT, Sharp K, et al. The UK Biobank resource with deep phenotyping and genomic data. Nature 2018; 562: 203–209.

Day FR, Loh PR, Scott RA, Ong KK, Perry JRB. A Robust Example of Collider Bias in a Genetic Association Study. Am J Hum Genet 2016; 98: 392–393.

Fang H, Knezevic B, Burnham KL, Knight JC. XGR software for enhanced interpretation of genomic summary data, illustrated by application to immunological traits. Genome Med 2016; 8: 129.

Fromer M, Roussos P, Sieberts SK, Johnson JS, Kavanagh DH, Perumal TM, et al. Gene expression elucidates functional impact of polygenic risk for schizophrenia. Nat Neurosci 2016; 19: 1442–1453.

de Leeuw CA, Mooij JM, Heskes T, Posthuma D. MAGMA: Generalized Gene-Set Analysis of GWAS Data. PLoS Comput Biol 2015; 11: e1004219.

Loh PR, Kichaev G, Gazal S, Schoech AP, Price AL. Mixed-model association for biobank-scale datasets. Nat Genet 2018; 50: 906–908.

Loh PR, Tucker G, Bulik-Sullivan BK, Vilhjálmsson BJ, Finucane HK, Salem RM, et al. Efficient Bayesian mixed-model analysis increases association power in large cohorts. Nat Genet 2015; 47: 284–290.

Ng B, White CC, Klein HU, Sieberts SK, McCabe C, Patrick E, et al. An xQTL map integrates the genetic architecture of the human brain's transcriptome and epigenome. Nat Neurosci 2017; 20: 1418–1426.

Qi T, Wu Y, Zeng J, Zhang F, Xue A, Jiang L, et al. Identifying gene targets for brain-related traits using transcriptomic and methylomic data from blood. Nat Commun 2018; 9: 2282.

Ramasamy A, Trabzuni D, Guelfi S, Varghese V, Smith C, Walker R, et al. Genetic variability in the regulation of gene expression in ten regions of the human brain. Nat Neurosci 2014; 17: 1418–1428.

Watanabe K, Taskesen E, Van Bochoven A, Posthuma D. Functional mapping and annotation of genetic associations with FUMA. Nat Commun 2017; 8: 1826.

Wiberg A, Ng M, Schmid AB, Smillie RW, Baskozos G, Holmes M V, et al. A genome-wide association analysis identifies 16 novel susceptibility loci for carpal tunnel syndrome. Nat Commun 2019; 10: 1030.

Zheng J, Erzurumluoglu AM, Elsworth BL, Kemp JP, Howe L, Haycock PC, et al. LD Hub: A centralized database and web interface to perform LD score regression that maximizes the potential of summary level GWAS data for SNP heritability and genetic correlation analysis. Bioinformatics 2017; 33: 272–279.

Supplementary Table 1. Summary of estimated heritability h^2 of the 3,144 IDPs (Elliott et al., 2018)

			mean	min	max
**Structural Imaging**	T1	Global volume (SIENAX)	0.41	0.32	0.45
		Subcortical volume (FIRST)	0.34	0.01	0.59
		Grey matter volume (FAST/FSL-VBM)	0.29	0.05	0.58
		Subcortical, white matter and CSF volume (FreeSurfer)	0.40	0.18	0.78
		Cortical area (FreeSurfer)	0.26	0.02	0.57
		Cortical Thickness (FreeSurfer)	0.20	0.00	0.38
		White matter hyperintensities volume	0.44	-	-
	T2*	Subcortical T2* value	0.32	0.08	0.62
**Diffusion Imaging**		TBSS-derived value	0.37	0.03	0.65
		Tractography-derived value	0.35	0.05	0.63
**Functional Imaging**	Task fMRI	Face-shape IDP	0.07	0.00	0.12
	rs-fMRI	Node amplitude (FSLnets)	0.19	0.00	0.36
	rs-fMRI	Functional connectivity edge (FSLnets)	0.05	0.00	0.30
	rs-fMRI	Functional connectivity edge – 6 ICs (ICA + FSLnets)	0.35	0.06	0.52

Supplementary Table 2. Significant associations between imaging-derived phenotypes (IDPs) and self-reported handedness. We examined all significant associations, Bonferroni-corrected for multiple comparisons across all IDPs (n=3,144). We identified numerous significant associations, almost exclusively using rfMRI connectivity analysis. **A. Detailed table of top 10 results.** They were all obtained using rfMRI connectivity measures (either using an ICA decomposition of the fMRI data into d=25 or d=100 independent components, "ICA25" and "ICA100" respectively), i.e. functional connectivity between a pair of networks identified by a single "edge" number. The most prevalent network in the most significant IDPs associated with handedness is the homolog of the language network in the right hemisphere (network 33 for ICA100, 21 for ICA25), encompassing Broca's area (BA44 and 45), the regions around the superior temporal sulcus, as well as premotor and primary motor regions centred around the tongue and mouth. The equivalent network is split in two in the left hemisphere at higher dimension (network 28 and 09 for ICA100, 13 for ICA25). Those main results can be summarised by, in left-handers: (i) a stronger connectivity between right and left language network (in bold), as well as (ii) a weaker connectivity between the right language network and the default-mode network (DMN) and salience network (in bold and italics). **B. Table of all significant results.** Results are ranked by effect size.

A.

rfMRI (ICA dimension; edge)	Networks pair	uncorr-p	functional connectivity	r	Effect in left-handers*	Identification of the pair of networks involved; hemisphere
ICA100; edge 524	28-33	5.2E-44	0.17	0.12	**Stronger functional connectivity**	**Language; L (mainly prefrontal, temporo-parietal)**<br>**Language; R**
ICA100; edge 505	09-33	4.1E-31	0.29	0.10	**Stronger functional connectivity**	**Language; L (mainly temporal)**<br>**Language; R**
ICA25; edge 7	01-05	2.7E-30	-0.71	0.10	Weaker functional connectivity	DMN<br>Fronto-parietal; R
ICA25; edge 203	13-21	4.8E-28	0.38	0.10	**Stronger functional connectivity**	**Language; L**<br>**Language; R**
ICA25; edge 191	01-21	1.9E-26	-0.27	0.09	***Weaker functional connectivity***	***DMN***<br>***Language; R***
ICA100; edge 509	13-33	7.1E-26	-0.68	0.09	***Weaker functional connectivity***	***Dorsal prefrontal (part of DMN)***<br>***Language; R***
ICA100; edge 525	29-33	5.1E-25	1.61	-0.09	***Weaker functional connectivity***	***Temporo-parietal junction (part of DMN)***<br>***Language: R***
ICA100; edge 138	02-18	1.6E-24	1.10	0.09	Stronger functional connectivity	Salience<br>Premotor and inferior parietal lobule; L
ICA100; edge 498	02-33	2.2E-19	0.28	-0.08	***Weaker functional connectivity***	***Salience***<br>***Language; R***
ICA25; edge 176	05-20	2.0E-18	0.82	-0.08	Weaker functional connectivity	Fronto-parietal; R<br>Precuneus and posterior intra-parietal sulcus (part of DMN)

*Determined by combining the values of functional connectivity (positive or negative partial correlation) with the corresponding r values (left-handedness coded as 2, right-handedness as 1, before confounds regression).

B.

IDP (measure)	IDP (location)	r	uncorr-p
rfMRI - connectivity	ICA100; edge 524	0.12	5.2E-44
rfMRI - connectivity	ICA100; edge 505	0.10	4.1E-31
rfMRI - connectivity	ICA25; edge 7	0.10	2.7E-30
rfMRI - connectivity	ICA25; edge 203	0.10	4.8E-28
rfMRI - connectivity	ICA25; edge 191	0.09	1.9E-26
rfMRI - connectivity	ICA100; edge 509	0.09	7.1E-26
rfMRI - connectivity	ICA100; edge 525	-0.09	5.1E-25
rfMRI - connectivity	ICA100; edge 138	0.09	1.6E-24
rfMRI - connectivity	ICA100; edge 498	-0.08	2.2E-19
rfMRI - connectivity	ICA25; edge 176	-0.08	2.0E-18
rfMRI - connectivity	ICA25; edge 72	-0.08	7.2E-18
rfMRI - connectivity	ICA25; edge 15	0.08	7.9E-18
rfMRI - connectivity	ICA100; edge 929	0.07	2.1E-17
rfMRI - connectivity	ICA25; edge 210	-0.07	6.8E-17
rfMRI - connectivity	ICA100; edge 60	0.07	9.3E-17
rfMRI - connectivity	ICA100; edge 165	-0.07	1.5E-16
rfMRI - connectivity	ICA100; edge 501	0.07	2.6E-16
rfMRI - connectivity	ICA100; edge 8	-0.07	8.2E-16
rfMRI - connectivity	ICA100; edge 663	-0.07	9.2E-16
rfMRI - connectivity	ICA100; edge 515	-0.07	2.8E-15
rfMRI - connectivity	ICA100; edge 360	-0.07	5.5E-15
rfMRI - connectivity	ICA100; edge 353	0.07	8.0E-15
rfMRI - connectivity	ICA100; edge 1107	0.07	2.2E-14
rfMRI - connectivity	ICA100; edge 522	0.07	6.5E-14
rfMRI - connectivity	ICA25; edge 206	-0.06	1.6E-13
rfMRI - connectivity	ICA25; edge 177	0.06	1.9E-13
rfMRI - connectivity	ICA100; edge 1140	0.06	2.3E-13
rfMRI - connectivity	ICA100; edge 103	-0.06	2.7E-13
rfMRI - connectivity	ICA100; edge 520	0.06	5.0E-13
rfMRI - connectivity	ICA25; edge 207	-0.06	1.1E-12
rfMRI - connectivity	ICA25; edge 110	-0.06	2.6E-12
rfMRI - connectivity	ICA25; edge 196	0.06	2.9E-12
rfMRI - connectivity	ICA100; edge 302	-0.06	3.5E-12
rfMRI - connectivity	ICA100; edge 518	-0.06	1.9E-11
rfMRI - connectivity	ICA100; edge 915	0.06	2.3E-11
rfMRI - connectivity	ICA25; edge 29	-0.06	5.5E-11
rfMRI - connectivity	ICA100; edge 628	-0.06	7.8E-11
rfMRI - connectivity	ICA100; edge 47	0.05	4.2E-10
rfMRI - connectivity	ICA100; edge 1161	-0.05	4.4E-10
rfMRI - connectivity	ICA100; edge 513	0.05	6.3E-10
rfMRI - connectivity	ICA100; edge 57	0.05	7.1E-10
rfMRI - connectivity	ICA100; edge 517	0.05	7.9E-10
rfMRI - amplitudes	ICA100; node 12	-0.05	1.1E-09
rfMRI - connectivity	ICA25; edge 114	0.05	1.4E-09
rfMRI - connectivity	ICA100; edge 377	-0.05	1.6E-09
rfMRI - connectivity	ICA100; edge 315	-0.05	1.7E-09
rfMRI - connectivity	ICA100; edge 325	0.05	4.2E-09
rfMRI - connectivity	ICA100; edge 201	0.05	4.6E-09
rfMRI - amplitudes	ICA25; node 13	-0.05	7.7E-09
rfMRI - connectivity	ICA25; edge 75	-0.05	2.9E-08
rfMRI - connectivity	ICA100; edge 759	-0.05	4.3E-08
rfMRI - connectivity	ICA100; edge 546	0.05	5.2E-08
rfMRI - connectivity	ICA100; edge 321	-0.05	6.6E-08
rfMRI - connectivity	ICA100; edge 222	-0.05	9.0E-08
rfMRI - connectivity	ICA100; edge 311	0.05	1.0E-07
rfMRI - connectivity	ICA100; edge 362	0.05	1.7E-07
rfMRI - connectivity	ICA100; edge 454	0.05	1.9E-07
rfMRI - connectivity	ICA100; edge 164	-0.05	2.3E-07
rfMRI - connectivity	ICA25; edge 11	-0.04	2.8E-07
rfMRI - connectivity	ICA25; edge 71	0.04	3.2E-07
rfMRI - connectivity	ICA100; edge 587	-0.04	5.2E-07
rfMRI - connectivity	ICA100; edge 606	-0.04	9.0E-07
rfMRI - connectivity	ICA25; edge 50	0.04	1.0E-06
rfMRI - connectivity	ICA100; edge 324	0.04	1.1E-06
rfMRI - connectivity	ICA100; edge 932	-0.04	1.8E-06
rfMRI - connectivity	ICA100; edge 337	0.04	2.0E-06
rfMRI - connectivity	ICA100; edge 731	-0.04	2.4E-06
rfMRI - connectivity	ICA100; edge 658	0.04	3.1E-06
dMRI - TBSS MO	External capsule; L	-0.04	3.6E-06
rfMRI - connectivity	ICA100; edge 309	-0.04	3.8E-06
rfMRI - connectivity	ICA25; edge 133	0.04	3.8E-06
rfMRI - connectivity	ICA25; edge 84	0.04	4.3E-06
rfMRI - connectivity	ICA100; edge 132	0.04	5.0E-06
rfMRI - connectivity	ICA100; edge 831	0.04	5.5E-06
rfMRI - amplitudes	ICA100; node 28	-0.04	5.7E-06
rfMRI - connectivity	ICA100; edge 594	-0.04	5.9E-06

Supplementary Table 3. eQTL of index SNPs at three associated loci. Statistically significant ($p<0.05$) eQTL for index SNP in different brain tissues, taken from GTEx v7 and BRAINEAC (see URLs). Brain-eMeta (Qi *et al.*, 2018) is a set of meta-analysed eQTL data from a meta-analysis of of GTEx, CMC (Fromer *et al.*, 2016), and ROSMAP (Ng *et al.*, 2017). Of the four index SNPs, rs199512 was associated with genes at a meta-analysis significant p-value of $p<5\times10^{-8}$.

CHROMOSOME	SNP	GENE	BRAIN TISSUES	SOURCE
**2**	rs13017199	*MAP2*	Occipital cortex	BRAINEAC
**6**	rs3094128	*MICB*	Cerebellar hemisphere	GTEx
**17**	rs199512	*MAPT*	Frontal cortex, hippocampus, occipital cortex, temporal cortex, thalamus	BRAINEAC
**17**	rs199512	*MAPT-AS1*	Nucleus accumbens, frontal cortex, cerebellum	GTEx
**17**	rs199512	*LRRC37A4P*	All	Brain-eMeta
**17**	rs199512	*RP11-798G7.6*	All	Brain-eMeta
**17**	rs199512	*RP11-798G7.8*	All	Brain-eMeta
**17**	rs199512	*MAPT*	All	Brain-eMeta
**17**	rs199512	*KANSL1*	All	Brain-eMeta
**17**	rs199512	*KANSL1-AS1*	All	Brain-eMeta
**17**	rs199512	*RP11-259G18.1*	All	Brain-eMeta
**17**	rs199512	*RP11-259G18.2*	All	Brain-eMeta
**17**	rs199512	*RP11-259G18.3*	All	Brain-eMeta
**17**	rs199512	*LRRC37A*	All	Brain-eMeta
**17**	rs199512	*LRRC37A2*	All	Brain-eMeta
**17**	rs199512	*LRRC37A3*	All	Brain-eMeta
**17**	rs199512	*WNT3*	All	Brain-eMeta
**17**	rs199512	*RP11-927P21.1*	All	Brain-eMeta

Supplementary Table 4. MAGMA Gene-Set Analysis. A MAGMA analysis was performed on the summary statistics in the left- vs right-handers GWAS. MAGMA gene-set analysis was performed for curated gene sets and GO terms obtained from MsigDB v6.1 (10655 gene sets - curated gene sets: 4738, GO terms: 5917). The top 10 significant gene sets with a Bonferroni-corrected p-value of < 0.05 are shown, and these have been ranked by numbers of genes overlapped.

Gene set	Number of genes overlapped	Beta	SE	p
GO_bp:go_neuron_projection_morphogenesis	383	0.153	0.0437	0.00024
GO_bp:go_cell_morphogenesis_involved_in_neuron_differentiation	351	0.169	0.0463	0.00013
GO_bp:go_neuron_migration	104	0.281	0.0875	0.00065
GO_bp:go_regulation_of_gliogenesis	88	0.305	0.0936	0.00057
Curated_gene_sets:kyng_environmental_stress_response_up	51	0.352	0.111	0.00073
Curated_gene_sets:smid_breast_cancer_relapse_in_lung_dn	37	0.461	0.125	0.00011
GO_bp:go_sympathetic_nervous_system_development	20	0.685	0.197	0.00025
Curated_gene_sets:reactome_sema3a_pak_dependent_axon_repulsion	13	0.745	0.215	0.00026
Curated_gene_sets:kyng_environmental_stress_response_not_by_uv_in_ws	12	0.857	0.262	0.00054
Curated_gene_sets:castellano_hras_and_nras_targets_dn	7	0.892	0.266	0.00040

Supplementary Table 5. Full results of SNP-based enrichment analysis with XGR. Table demonstrating the results of SNP-based enrichment analysis using 4,007 SNPs in the left- vs right-handers GWAS with a p-value suggestive of association ($p<5\times10^{-5}$). The ontologies are ranked by False Discovery Rate, and the table also shows the Z-score, p-value and the number of SNPs overlapped with each ontological term.

Term Name	Z-score	p	False Discovery Rate	SNPs overlapped
Parkinson's disease	26.5	6.60E-21	2.60E-19	12
neurodegenerative disease	11.6	1.40E-13	2.80E-12	15
intra cranial volume	35	2.00E-11	2.60E-10	3
Miscellaneous movement disorder due to genetic neurodegenerative disease	19.4	7.10E-09	5.50E-08	3
Frontotemporal neurodegeneration with movement disorder	19.4	7.10E-09	5.50E-08	3
Rare genetic movement disorder	17.8	1.50E-08	9.80E-08	3
movement disorder	17.4	1.90E-08	1.00E-07	3
Corticobasal degeneration	21.2	5.80E-08	2.80E-07	2
Rare genetic neurological disorder	10.5	0.0000011	0.0000048	3
genetic disorder	6.56	0.0000078	0.00003	5
multiple system atrophy	9.86	0.0000083	0.00003	2
brain volume measurement	7.2	0.000018	0.000059	3
Atrophy	6.4	0.000099	0.0003	2
ovarian neoplasm	5.75	0.00018	0.00046	2
ovarian carcinoma	5.75	0.00018	0.00046	2
ovarian disease	4.88	0.00042	0.001	2
celiac disease	4.52	0.00061	0.0014	2
brain measurement	3.52	0.0016	0.0035	3
tauopathy	3.18	0.0024	0.0049	4
bone density	2.8	0.0052	0.01	2
bone fracture related measurement	2.7	0.006	0.011	2
bone measurement	2.17	0.013	0.023	2
Alzheimer’s disease	2.18	0.014	0.023	3
inflammatory bowel disease	2.1	0.015	0.025	3
urogenital neoplasm	1.66	0.028	0.044	2
body mass index	1.6	0.035	0.052	3
digestive system disease	1.57	0.039	0.057	4
skin disease	1.31	0.049	0.068	2
body weights and measures	1.36	0.058	0.079	5
reproductive system disease	1.15	0.063	0.082	2
metabolic disease	0.783	0.11	0.14	2
lung disease	0.586	0.15	0.18	2
schizophrenia	0.499	0.17	0.2	2
epithelial neoplasm	0.405	0.19	0.21	2
carcinoma	0.405	0.19	0.21	2
autoimmune disease	0.297	0.25	0.27	3
cancer	-0.179	0.4	0.41	2
respiratory system disease	-0.193	0.4	0.41	2
neoplasm	-0.34	0.47	0.47	2

Supplementary Table 6. Genetic correlation results between handedness and 14 neuro-psychiatric phenotypes executed in LDHub. GWAS summary statistics from the left- vs right-handers GWAS were compared against the GWAS summary statistics available on LDHub for neurological and psychiatric traits. Each row of the table demonstrates the two phenotypes being correlated, the PMID for the relevant GWAS, the trait category, the ethnicity of the participants in the relevant GWAS, correlation coefficient (r_g), the direction of the correlation between left-handedness and the trait in question, standard error (se), z-sore (z) and p-value. Results are ranked by p-value.

Trait 1	Trait 2	PMID	Category	Ethnicity	r_g	Direction	se	z	p
Left-handedness	Schizophrenia	25056061	psychiatric	Mixed	0.1324	+	0.0429	3.0828	0.0021
Left-handedness	Parkinson's disease	19915575	neurological	European	-0.2379	–	0.0884	-2.6915	0.0071
Left-handedness	Anorexia Nervosa	24514567	psychiatric	European	0.1504	+	0.059	2.5512	0.0107
Left-handedness	Bipolar disorder	21926972	psychiatric	European	0.1548	+	0.0691	2.2415	0.025
Left-handedness	PGC cross-disorder analysis	23453885	psychiatric	European	0.1296	+	0.0644	2.0115	0.0443
Left-handedness	Alzheimer's disease	24162737	neurological	European	-0.186	–	0.1148	-1.6209	0.105
Left-handedness	Subjective well being	27089181	psychiatric	European	-0.1176	–	0.0741	-1.5874	0.1124
Left-handedness	Autism spectrum disorder	N/A	psychiatric	European	0.0997	+	0.0809	1.2328	0.2177
Left-handedness	Attention deficit hyperactivity disorder (GC)	27663945	psychiatric	European	0.1371	+	0.1684	0.8142	0.4156
Left-handedness	Attention deficit hyperactivity disorder (No GC)	27663945	psychiatric	European	0.1354	+	0.1677	0.8076	0.4193
Left-handedness	Major depressive disorder	22472876	psychiatric	European	0.0686	+	0.097	0.7075	0.4792
Left-handedness	Depressive symptoms	27089181	psychiatric	European	0.0161	+	0.0627	0.2568	0.7973
Left-handedness	Attention deficit hyperactivity disorder	20732625	psychiatric	European	-0.0196	–	0.1371	-0.1428	0.8865
Left-handedness	Amyotrophic lateral sclerosis	27455348	neurological	European	0.0142	+	0.1234	0.1154	0.9081

Supplementary Table 7. Significant associations between imaging-derived phenotypes (IDPs) and loci genome-wide (GW) associated with handedness. We examined all significant associations, Bonferroni-corrected for multiple comparisons across all IDPs (n=3,144) and all GW significant loci (n=4). We identified several significant associations only for one of the four loci (rs199512), especially in white matter tracts using diffusion MRI (dMRI) measures, and more particularly in the "superior longitudinal fasciculus" (in bold). Results are ranked by effect size.

IDP (measure)	IDP (location; hemisphere)	beta	uncorr-p
dMRI - TBSS L3	Anterior limb of internal capsule; R	-0.100	3.0E-09
dMRI - TBSS L1	**Superior longitudinal fasciculus; R**	-0.100	3.4E-09
dMRI - TBSS OD	**Superior longitudinal fasciculus; R**	0.100	5.5E-08
dMRI - TBSS OD	**Superior longitudinal fasciculus; L**	0.099	2.5E-07
dMRI - TBSS L3	Anterior limb of internal capsule; L	-0.098	9.8E-09
dMRI - TBSS ICVF	Superior fronto-occipital fasciculus; R	0.098	1.4E-08
dMRI - TBSS OD	Posterior corona radiata; R	0.092	1.4E-07
dMRI - ProbtrackX ICVF	Forceps minor	0.092	7.7E-07
dMRI - ProbtrackX L1	**Superior longitudinal fasciculus; R**	-0.091	1.5E-08
dMRI - TBSS ICVF	Anterior corona radiata; R	0.09	5.3E-07
dMRI - TBSS L1	**Superior longitudinal fasciculus; L**	-0.09	5.4E-07
dMRI - TBSS ICVF	Anterior corona radiata; L	0.09	6.7E-07
dMRI - TBSS ICVF	Cingulum bundle; R	0.09	8.2E-07
dMRI - TBSS FA	Anterior limb of the internal capsule; L	0.09	1.7E-06
dMRI - TBSS ICVF	Superior fronto-occipital fasciculus; L	0.088	4.0E-07
dMRI - ProbtrackX MD	**Superior longitudinal fasciculus; R**	-0.086	7.1E-07
dMRI - TBSS L1	External capsule; R	-0.083	1.8E-06
dMRI - TBSS MD	Superior fronto-occipital fasciculus; L	-0.079	1.9E-06
dMRI - TBSS L1	Superior corona radiata; R	-0.078	1.8E-06
FreeSurfer (DKT atlas)	Lateraloccipital area; R	0.077	1.5E-07
FreeSurfer (DKT atlas)	Lateraloccipital area; L	0.074	4.8E-07
dMRI - ProbtrackX L1	Superior thalamic radiations; R	-0.073	5.6E-07
dMRI - ProbtrackX MD	Superior thalamic radiations; R	-0.072	8.3E-07
FreeSurfer (DKT atlas)	Fusiform area; R	0.069	4.0E-07
FreeSurfer (DKT atlas)	Fusiform area; L	0.069	7.6E-07
FreeSurfer (volume)	Ventral diencephalon	0.062	1.1E-06

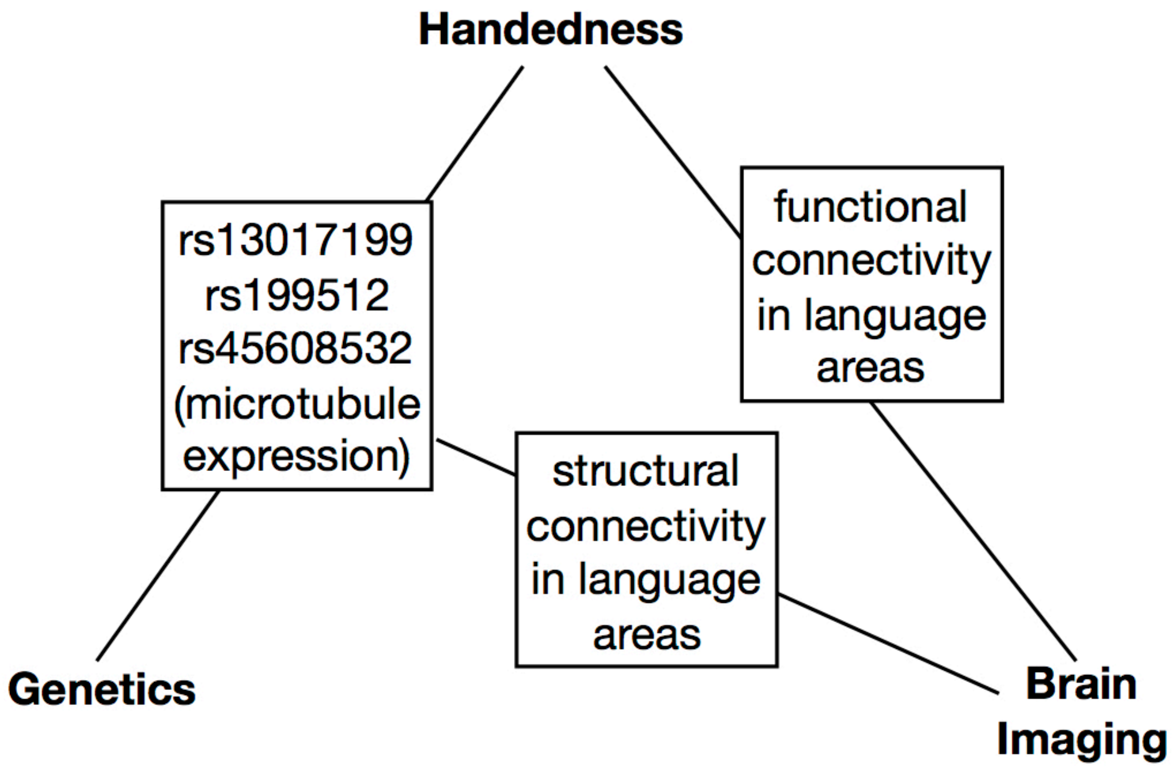

Supplementary Figure 1. Summary of results from the genetics-handedness, genetics-brain imaging, and handedness-brain imaging studies.

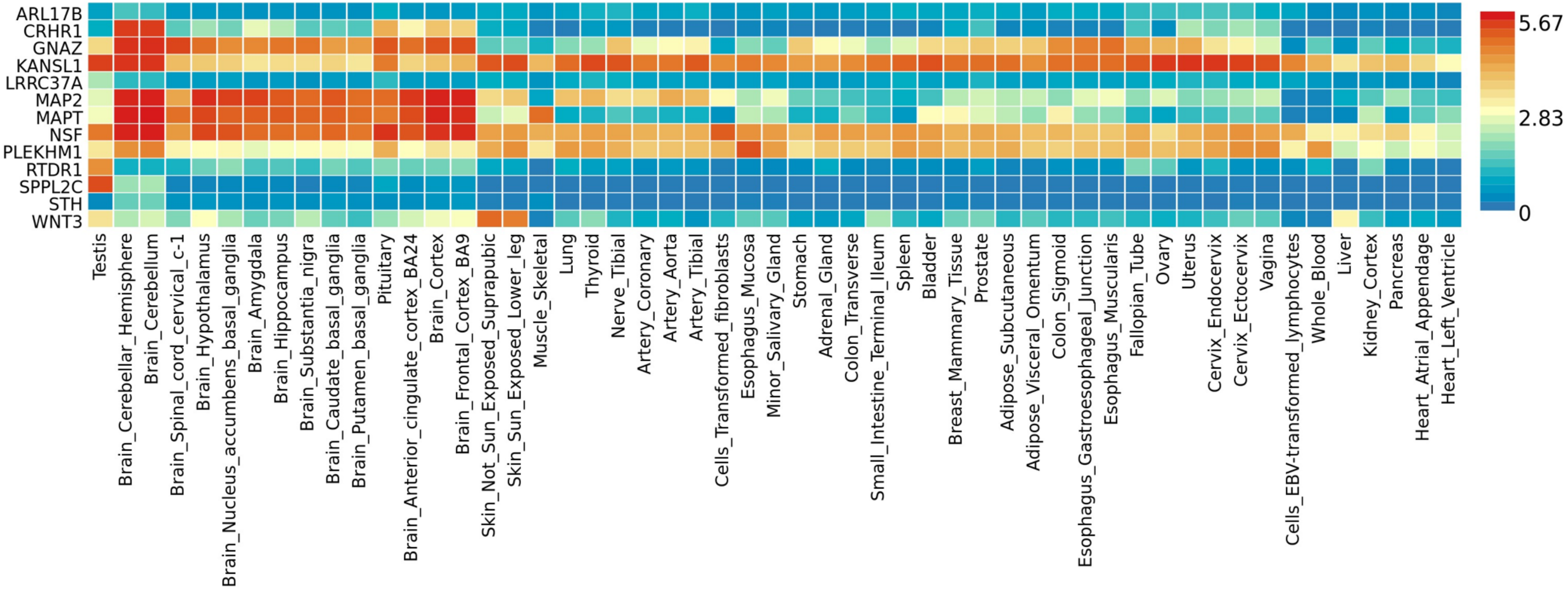

Supplementary Figure 2. Heat map of gene expression across 53 tissue types. This analysis was implemented in FUMA, and demonstrates the average expression values of the 13 genes positionally mapped by FUMA in the left- vs right-handers GWAS, across 53 tissue types in GTEx v7. This is an averaged expression value per tissue type per gene following winsorization at 50 and log 2 transformation with pseudocount 1. The expression value is in Transcripts per Million, and genes have been organised by hierarchical clustering.
